# Familial Case of Pelizaeus-Merzbacher Disorder Detected by Oligoarray Comparative Genomic Hybridization: Genotype-to-Phenotype Diagnosis

**DOI:** 10.1155/2017/2706098

**Published:** 2017-01-04

**Authors:** Kimia Najafi, Roxana Kariminejad, Kaveh Hosseini, Azadeh Moshtagh, Gole Maryam Abbassi, Neda Sadatian, Masood Bazrgar, Ariana Kariminejad, Mohamad Hassan Kariminejad

**Affiliations:** ^1^Kariminejad-Najmabadi Pathology and Genetics Center, Tehran, Iran; ^2^Tehran Heart Center, Tehran University of Medical Sciences, Tehran, Iran

## Abstract

*Introduction*. Pelizaeus-Merzbacher disease (PMD) is an X-linked recessive hypomyelinating leukodystrophy characterized by nystagmus, spastic quadriplegia, ataxia, and developmental delay. It is caused by mutation in the PLP1 gene.* Case Description*. We report a 9-year-old boy referred for oligoarray comparative genomic hybridization (OA-CGH) because of intellectual delay, seizures, microcephaly, nystagmus, and spastic paraplegia. Similar clinical findings were reported in his older brother and maternal uncle. Both parents had normal phenotypes. OA-CGH was performed and a 436 Kb duplication was detected and the diagnosis of PMD was made. The mother was carrier of this 436 Kb duplication.* Conclusion*. Clinical presentation has been accepted as being the mainstay of diagnosis for most conditions. However, recent developments in genetic diagnosis have shown that, in many congenital and sporadic disorders lacking specific phenotypic manifestations, a genotype-to-phenotype approach can be conclusive. In this case, a diagnosis was reached by universal genomic testing, namely, whole genomic array.

## 1. Introduction

Pelizaeus-Merzbacher Disorder (PMD) (OMIM 312080) is a rare central nervous disorder caused by a defect in myelination. It is therefore classified as leukodystrophy and is inherited as an X-linked disorder caused by a defect in myelination. Its cardinal features are nystagmus, spastic quadriplegia, ataxia, and developmental delay [1]. PMD is an early onset neurological disorder [2].

The most common cause of PMD is a mutation of the Proteolipid protein (PLP) gene producing a structural protein involved in the construction of central nervous system myelin [3–6].

PLP is located on the long arm of the X-chromosome (Xq21-22).

However, it is known that deletion or duplication of the region including this gene [3] can lead to the same phenotype in males. The majority of cases are caused by PLP1 duplication [3]. Inoue et al., 1996, suggest that PLP gene duplication plays an important role in myelin abnormality and hence PMD [2]. PMD is an early onset neurological disorder in which coordination, motor function, and intellectual ability all are delayed [3].

Renier et al., 1981, described 3 types of PMD: the classic type, which is characterized by nystagmus, ataxia, microcephaly, and abnormal somatic development with onset at infancy; the conatal type with rapid progression and early death; and the transitional form which is intermediate [7]. Mimault et al., 1999, reported 82 sporadic cases of PMD, where PLP gene duplication was the most frequent abnormality detected in 62% of cases [8]. It is hypothesized that deletion is infrequent since bigger deletions will result in infertility as well [9].

PMD is usually suspected by the presence of typical neurological findings in physical examination and abnormal myelination in Magnetic Resonance Imaging (MRI). Less than 1% can be detected by cytogenetic studies [10].

## 2. Methods

Blood samples were collected in EDTA tubes. We performed chromosomal microarray on DNA extracted from EDTA blood by salting out procedure [11]. Whole genome oligoarray comparative genomic hybridization (OA-CGH) was performed using CYTOCHIP ISCA 8 × 60 K version 2 per manufacturer protocol. Images were scanned using Innopsys 910 and was analyzed using BlueFuse Multi Software version 3. The array consists of 60000 spots with average backbone resolution of 51 kbs and close to 500 targeted disease regions. The sample was hybridized twice against male and female samples used as reference.

Parents gave their informed consent to participate in this study and publish their family trees and family data. The study was approved by the Kariminejad-Najmabadi Pathology & Genetics ethics committee.

## 3. Case Report

**Figure 1:**
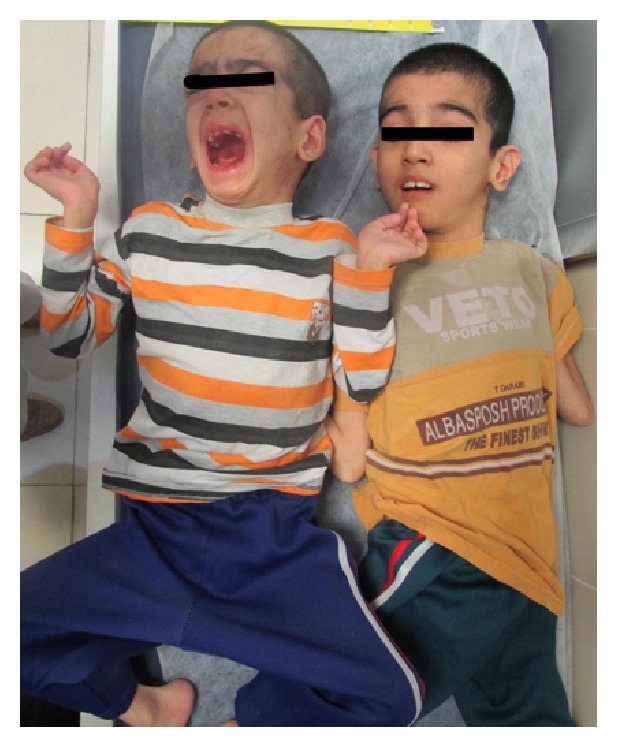
Two offsprings of the family, with Pelizaeus-Merzbacher Disorder. Note spastic posturing.

**Figure 2:**
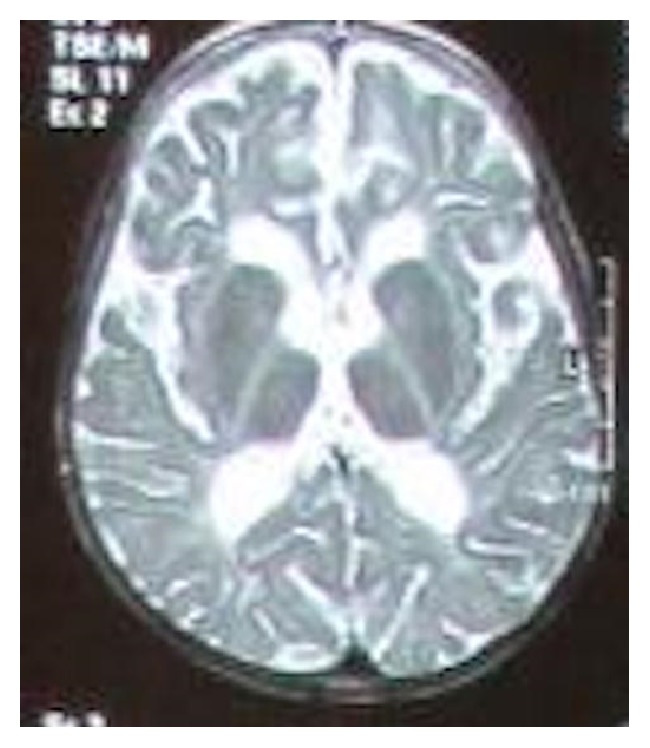
Brain MRI of the patient shows cerebral atrophy. Note hypomyelination and leukodystrophy.

Here we present a 9-year-old Iranian boy (Figure 1) to unrelated apparently healthy parents. He was referred to Kariminejad-Najmabadi Pathology & Genetics Center for genetic counseling. He had severe mental retardation according to WHO ICD 10 guideline with severe developmental delay. He could not walk, talk, or even hold his neck. He had a history of seizures since he was 4 and was being managed with phenobarbital. He was cachectic. At physical examination bilateral nystagmus, microcephaly, spastic quadriplegia, and macroorchidism were noted. Chromosomal study had been previously performed and reported as normal. Molecular testing for Fragile-X was normal. Brain MRI reported cerebral atrophy (Figure 2). He had an older brother aged 15 years (Figure 1) and a maternal uncle with similar clinical features.

Oligoarray CGH was performed for both affected offspring and the mother.

## 4. Results

**Figure 3:**
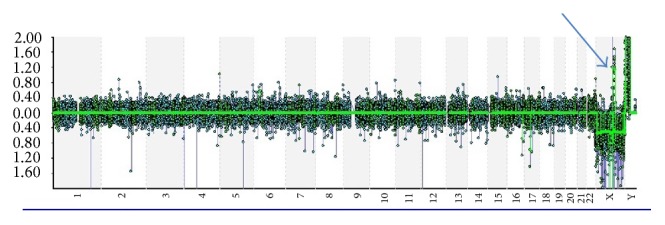
Oligoarray CGH results. Note the arrow shows duplication on X-chromosome.

Whole genome oligoarray CGH was performed. Results of hybridization for both offspring and mother showed a gain of 436 Kb on Xq22.2 from nucleotide 102,731,413 to nucleotide 103,167,741 (Figure 3).

This region includes these 4 OMIM genes:

Transcription elongation factor a-like 1: TCEAL1 (^*∗*^300237), not associated with any disease.
Mortality factor 4-like protein 2: MORF4L2 (^*∗*^300409), not associated with any disease.
Proteolipid protein 1: PLP1 (^*∗*^300401).
Ras-associated protein 9B: RAB9B (^*∗*^300285), not associated with any disease.

## 5. Discussion

According to the classification used by Renier et al., 1981 (the classic type, the conatal type, and the transitional form), our patient has the classic PMD. The phenotypic findings of this type include nystagmus, ataxia, microcephaly, and abnormal somatic development with onset at infancy. All features were present in both affected males in our pedigree.

The clinical features seen in most patients diagnosed with PMD, based on different articles, are developmental delay, microcephaly, hearing impairment, rotary head movements, ataxia, spasticity, psychomotor delay, scanning speech, and dysmyelination of the brain [5, 7, 12]. Our patients have developmental delay, microcephaly, spasticity, psychomotor delay, and dysmyelination of the brain.

All these findings are rather common manifestations in many syndromic and nonsyndromic types of intellectual delay and therefore extremely insubstantial for reaching a clinical diagnosis.

For example, Alexander disease is a rare neurological disease with defect in myelination and it is known by seizure, developmental delay, intellectual disability, and spasticity, similar to PMD; however it has megalencephaly which is absent in PMD. Alexander disease is inherited in autosomal dominant fashion [13] which is not compatible with our family tree.

Leukoencephalopathy with vanishing white matter can be considered in the differential diagnosis of PMD. It is an autosomal recessive disease with neurological features such as spasticity, developmental delay, microcephaly, seizure, and optic atrophy. All features are similar to this PMD except optic atrophy and MRI findings [14].

MRI findings with advanced disease reveal a diffuse cerebral hemispheric leukoencephalopathy in which increasing areas of the abnormal white matter have a signal intensity close to that of CSF on all pulse sequence while in PMD we have hypomyelination. Arts syndrome is another disease characterized with developmental delay, intellectual impairment, nystagmus, quadriplegia, and seizure with X-linked inheritance pattern; however it is an enzymopathy, which causes hypotonia and sensorineural deafness, which are absent in PMD [15].

Another differential diagnosis for PMD is adrenoleukodystrophy. It is a progressive neurodegenerative disorder with X-linked inheritance. It is known by developmental delay, seizure, and spasticity as seen in PMD, but in adrenoleukodystrophy adrenal glands are affected and patients have adrenal insufficiency [16].

We used the approach recommended by The International Standards for Cytogenomics Array Consortium of the use of chromosomal microarray analysis as a first-tier testing for diagnosis of intellectual/developmental delay. The PLP duplication was detected in the proband by whole genome array. Familial study including microarray analysis of affected brother and mother and the presence of the same genomic imbalance in both substantiated the diagnosis and the role of the duplication in the etiology of the phenotype. Phenotype-to-genotype correlation was indicated.

Considering the affected siblings and uncle, it was apparent that we have a familial form of PMD where the carrier female mother is unaffected. Carrier females with duplication in PLP gene are usually asymptomatic [12]. Both affected offspring had similar features and showed the phenotypic manifestations of the disease; however, the MRI when first seen by radiologist did not report any leukodystrophy.

Consultation with specialists in white matter and hypomyelination disorders confirmed the presence of hypomyelination and leukodystrophy which was expected in a classical PMD. We believe that this case emphasizes the importance of a systematic genotype-to-phenotype approach in clinical diagnosis of genetic disorders without very specific distinguishing phenotypic manifestations.

PMD is a rare neurological disorder with no specific and pathognomonic clinical feature and is mostly diagnosed by high T2 signal intensity throughout the brain in MRI. This case was referred to as unknown case of mental retardation; however the exact etiology was established after precise genetic tests.

## 6. Conclusion

This case highlights the importance of step-by-step genetic testing to reach a definite diagnosis for cases without specific clinical findings. In this case, first step was karyotyping, next was Fragile-X study, and, after normal results, OA-CGH was performed. Radiological studies should have detected the hypomyelinating leukodystrophy but was missed by radiologists without expertise in white matter disorders.
